# Design Parametrization of Central Venous Catheters for Pediatric Dialysis: Supporting the Quest for the Most Influential Features

**DOI:** 10.1097/MAT.0000000000002547

**Published:** 2025-09-05

**Authors:** Claudia Bruno, Rukshana Shroff, Silvia Schievano, Claudio Capelli

**Affiliations:** From the * University College London – Institute of Child Health, London, United Kingdom; † Department of Nephrology, Great Ormond Street Hospital for Children, London, United Kingdom; ‡ University College London – Institute of Cardiovascular Science, London, United Kingdom.

**Keywords:** central venous catheters, hemodialysis, design, computational fluid dynamics, geometrical features, pediatric

## Abstract

Pediatric hemodialysis is a life-saving treatment for children with chronic kidney diseases. Central venous catheters (CVCs) are the most commonly used vascular access, despite being commonly subject to complications leading to inadequate hemodialysis and catheter replacement. The available CVCs feature various design elements reflecting ongoing efforts to achieve optimal performance. Computational fluid dynamics (CFD) can contribute to analyze the flow dynamics within the CVCs. The aim of this study is to investigate the design parameters that most influence the flow performance of CVCs. A design of experiment (DOE) was set up to assess the CFD of two CVC models of 6.5F and 8F size. Blood flow rates, shear stress, residence time, and platelet lysis index were evaluated. The results showed how the proximal side holes were the most influential geometrical features, influencing both the flow rates (*r* > 0.64) and the shear stress of the CVCs (|*r*| > 0.5). At increased flow rate, the side holes were found to be competing with the tip in terms, especially, of residence time inside the CVC. The findings of this DOE show how CFD can contribute to understand the influence of design parameters and potentially guide the development of optimized pediatric-specific CVC models.

## Graphical abstract

**Figure:**
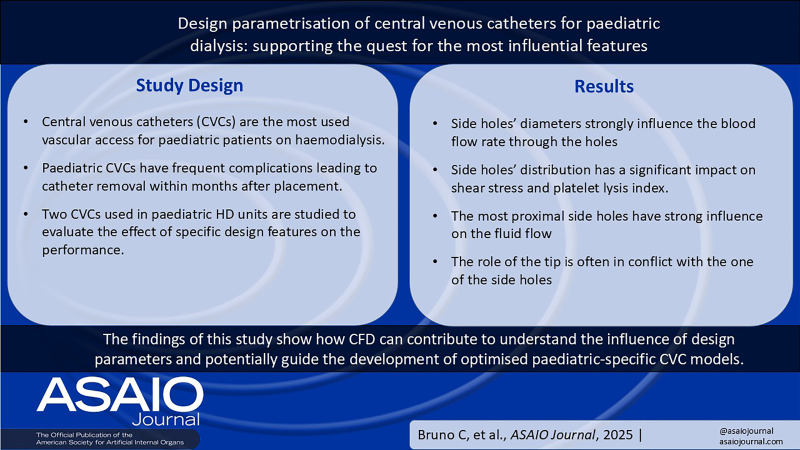

Pediatric end-stage renal disease requires kidney replacement treatments in the form of transplant or dialysis.^1^

In Europe, the incidence of patients younger than 15 years of age starting kidney replacement therapy ranges between 5.5 and 6.6 per million age-related population per year.^2^ For almost 50% of patients, the first treatment is hemodialysis (HD),^2^ alternative treatments including peritoneal dialysis and pre-emptive kidney transplant. The “Achille’s heel” of HD is the vascular access, accounting for significant morbidity and even mortality.^3^ Achieving pediatric HD vascular access is complex and maintaining it is challenging.^4^ Central venous catheters (CVCs) are the most commonly used pediatric vascular access. Central venous catheters are small tubes made of soft polymers (*e.g*., polyurethane or silicon) usually placed in the superior vena cava (SVC), with the tips laying in the right atrium. Central venous catheters include a blood-withdrawing tip (arterial) and a cleansed blood-returning tip (venous) which allows to perform the HD in continuous flow. The design and position of the arterial and venous tip should always prevent blood recirculation (cleansed blood re-entering the HD circuit). The design of CVCs for HD varies depending on the manufacturer but the main features generally are: lumen (single or double); tip; one or more side openings (holes or slots).^5,6^ Almost half of the CVCs of patients treated in pediatric HD units around the globe require replacement.^7^ Central venous catheter’s most common complications include thrombosis and fibrin sheath obstruction, leading to insufficient blood flow and inadequate HD.^7–9^ In 11% of cases, these malfunctions occur within 1 month after CVC placement.^7^

Computational studies have previously investigated the fluid-dynamic performance of existing CVC designs,^10–15^ the role of the side holes^16^ and suggested new designs for HD catheters.^17,18^ Despite the potential of computational methods, a systematic approach to improve specifically the design and the function of pediatric CVC still lacks.

The aim of this study is thus to investigate all the design parameters that may influence the performance of CVCs by means of a design of experiments (DOE) which consider a large number of different designs using sampling methods. The findings of this analysis can guide the development of new optimized pediatric-specific models.

## Methods

Two models resembling commercially available CVCs were included in this analysis:

- Tesio 6.5F (MedCOMP, Harleysville, PA): a single-lumen dual line with a cut straight tip hole and six side holes arranged in spiral configuration in proximity of the tip.
- Hemo-Cath 8F (MedCOMP): a double-lumen line with step tip hole and a circle-C arterial lumen profile. The tip of the venous lumen includes two opposite side holes. The tip of the arterial lumen has a parallel dual-hole configuration.

**Figure 1.**
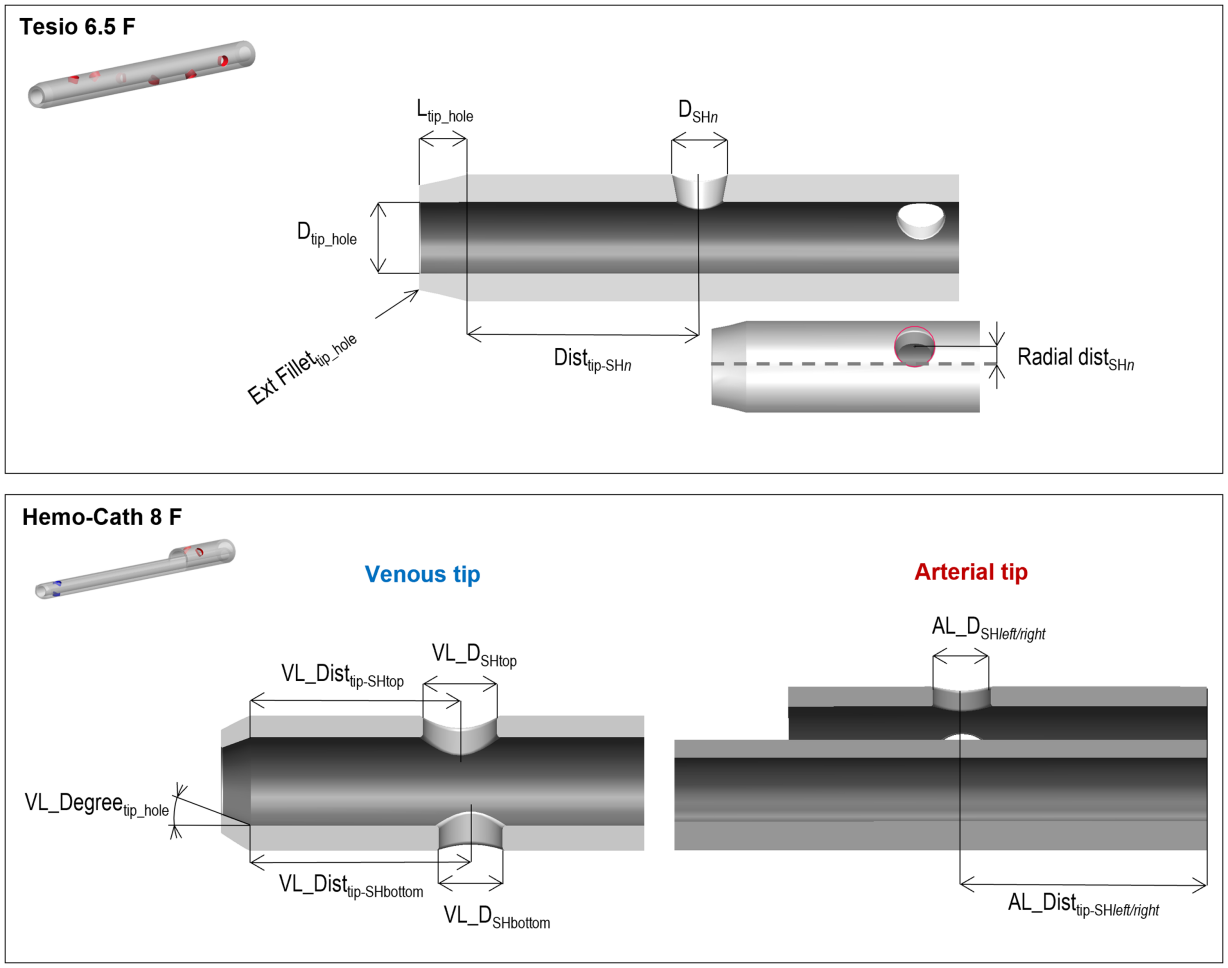
Geometry of the two CVCs with the labeled design variables. The Tesio 6.5F model is formed by two identical lumens; only one of them is shown here. CVC, central venous catheter.

Computer-aided design (CAD) models of the catheters were created in SolidWorks (Dassault Systèmes SE, Vélizy-Villacoublay, France) and the dimensions of the design variables were labeled (Figure 1) before the geometrical parametrization which was carried out with Ansys DesignModeler (Canonsburg, PA).

### Design of Experiments

A DOE was set up to i) identify the input parameters which have the strongest influence on the fluid-dynamic outputs; and ii) investigate possible interactions among them. The exploration designs were evaluated by the computational fluid dynamics (CFD) solver, and for each design, the corresponding response values were obtained.

The DOE was run in optiSLang (Ansys, Canonsburg, PA). To derive high-accuracy results with a low number of samples, Latin Hypercube Sampling was chosen to locate the sampling points. A sample size of minimum 10 times the number of the input variables was considered accurate to model the entire design space. For each sampling point, a set of input parameters was sampled by means of optiSLang and processed within Ansys Workbench for updating geometry and mesh before CFD analysis. Two additional scripts (optiSLang Python v3.7.13) were implemented to filter out geometries where side holes were overlapping and to label correctly the holes based on their axial distance from the tip hole.

The accuracy of the sampling was verified by comparing the mean values of the samples with the mean value of the requested distribution range for each input variable.

### Geometrical Parametrization and Sampling Strategy

The CAD files of the CVC models were imported into Ansys DesignModeler and geometrical features were defined as input parameters of the DOE.

The Tesio 6.5F model included 21 design variables (*i.e*., input variables):

- Diameter (*D*_SHn_) of each side hole (n = 1–6)
- Position of each side hole defined as the combinations of the axial distance from the tip hole (Dist_tip-SHn_) and the radial distance from the axis of the catheter (Radial_Dist_SHn_)
- Shape and size of the tip defined by its diameter (*D*_tip_hole_) and length (*L*_tip_hole_) together with the degree of the external fillet (Ext Fillet_tip_hole_).

All input variables were continuous except for the radial distances which were discrete (0, 2.38, 4.75 mm) to have a maximum of three holes equally spaced around the CVC body.

The Hemo-Cath 8F model included nine input variables, with four identified on the arterial lumen and five on the venous lumen:

- Diameter (AL_*D*_SH left/right_) of each arterial side hole
- Position of each arterial side hole defined as the axial distance from the entrance of the tip (AL_Dist_tip-SH left/right_)
- Diameter (VL_*D*_SH top/bottom_) of each venous side hole
- Position (VL_Dist_tip-SH top/bottom_) of each side hole defined as the axial distance from the tip hole (VL_Dist_tip-SH top/bottom_)
- Size of the venous tip defined as the degree of opening of the tip hole (VL_Degree_tip_hole_).

All input parameters were continuous.

All input parameters for each CVC model are reported with their ranges in Table 1.

**Table 1.** Input Parameters and Their Range for the Tesio 6.5F Models (Left) and for the Hemo-Cath 8F Models (Right)

Input Geometrical Parameters							
Tesio 6.5F				Hemo-Cath 8F			
Name	Reference Value (mm)	Range (mm)		Name	Reference Value (mm)	Range (mm)	
		Min	Max			Min	Max
Arterial lumen				Arterial lumen			
SH diameter	1	0.6	1.1	SH diameter (left/right)	1.02	0.6	1.2
Axial distance SH1	23.97	4	27.5	Axial distance SH	7	2	8
Axial distance SH2	20.01	4	25.4	Venous lumen			
Axial distance SH3	16.05	4	23.3	SH top diameter	1	0.6	1
Axial distance SH4	12.09	2	21.2	SH bottom diameter	0.86	0.6	1
Axial distance SH5	8.13	2	19.1	Axial distance SH top	2.825	1.5	24
Axial distance SH6	4.17	2	8.5	Axial distance SH bottom	2.965	1.5	27
Length of the tip hole	0.86	0.86	6	Opening degree of the tip hole	0.02°	0.02°	30°
Internal diameter of the tip hole	1.27	0.4	1.27				
External fillet of the tip hole	0.03	0.03	0.5				
	**Reference Value (mm**)	**Tested Positions (mm**)					
Radial distance SH1	5.40	0; 2.38; 4.75					
Radial distance SH2	4.32	0; 2.38; 4.75					
Radial distance SH3	3.24	0; 2.38; 4.75					
Radial distance SH4	2.16	0; 2.38; 4.75					
Radial distance SH5	1.08	0; 2.38; 4.75					
Radial distance SH6	0	0; 2.38; 4.75					

The radial distance of the 6.5F model has been discretized to take into account possible physical constraints.

SH, side hole.

### Computational Fluid Dynamics Analyses and Model Responses

Each generated model of CVCs was coaxially placed inside a straight cylinder representing an idealized geometry of SVC of different patient groups. Superior vena cava diameters were 6 and 8.5 mm for Tesio 6.5F– and Hemo-Cath 8F–derived models, respectively. Superior vena cava length was 50 cm for Tesio 6.5F and 60 cm for Hemo-Cath 8F ensuring a fully developed velocity profile. The fluid domains were meshed in Ansys Meshing module using >2M tetrahedral elements.

Each model was tested at three different flow conditions resembling the spectrum of working conditions in clinics. Since the arterial lumen is known for being the most critical one,^12^ this was the only one simulated for the single-lumen 6.5F models. The 6.5F models were tested at 20, 30, and 40 ml/min. The 8F models were simulated at 50, 60, and 70 ml/min. A flat velocity profile was imposed at the inlet of the SVC to match physiological flow rates for the two patient groups: 810 and 1,450 ml/min. A zero reference pressure was set at the outlet of the SVC models. Walls were rigid, and a no-slip condition was applied with steady state simulations carried out under the assumption of laminar flow. The blood was modeled as an incompressible Newtonian fluid with a constant viscosity (0.0035 Pa·s) and density of 1,060 kg/m^3^. Navier-Stokes equations were resolved within ANSYS Fluent (Canonsburg, PA) using a pressure-based solver with couple scheme for pressure velocity coupling, and least squares cell-based, second order, and second-order upwind for gradient, pressure, and momentum, respectively. The equations for the platelet lysis index and the residence time (RT) were resolved using first-order upwind scheme. All simulations ran in batch mode on a workstation with Intel Xeon W-2275 CPU (3.30 GHz) processor and 64.0 GB of RAM.

Four parameters of interest for the DOE were computed to evaluate the catheters’ performance: platelet lysis index (PLI_l_),^19^ shear stress (SS),^12^ RT,^20^ and blood flow rates through the openings of the CVC.

All the parameters (maximum and average values) were evaluated at the volume containing the tip of the catheters where the blood exchange takes place. The 6.5F models’ response resulted in 13 output parameters on the arterial lumen. The 8F models’ response was analyzed on both the venous and arterial lumen resulting in a total of 18 output parameters, nine on each lumen. Computational fluid dynamics results were collected within optiSLang and postprocessed using Python scripts created to investigate the responses of the sampling points and the interaction between the design parameters.

### Statistical Analysis

Spearman’s rank correlation coefficients were computed to identify associations between design parameters and model responses, estimate the strength of these relationships, and to assess positive or negative correlations. Correlation coefficients were displayed in correlation matrices only if they were above a threshold of 0.50. Correlations were considered very strong if higher than 0.90, strong if included between 0.89 and 0.70, and moderate between 0.69 and 0.40. All correlation coefficients below 0.39 were considered weak or negligible. The correlation coefficients were computed in Python using Pandas v1.4.4 library. Kruskal-Wallis test (or one-way analysis of variance) was used to analyze the difference among unpaired and nonparametric data. The statistical tests were carried out in Python using SciPy v1.13.1 library. Significance was set at *p* value <0.05.

## Results

### Geometrical Parametrization and Sampling Strategy

**Figure 2.**
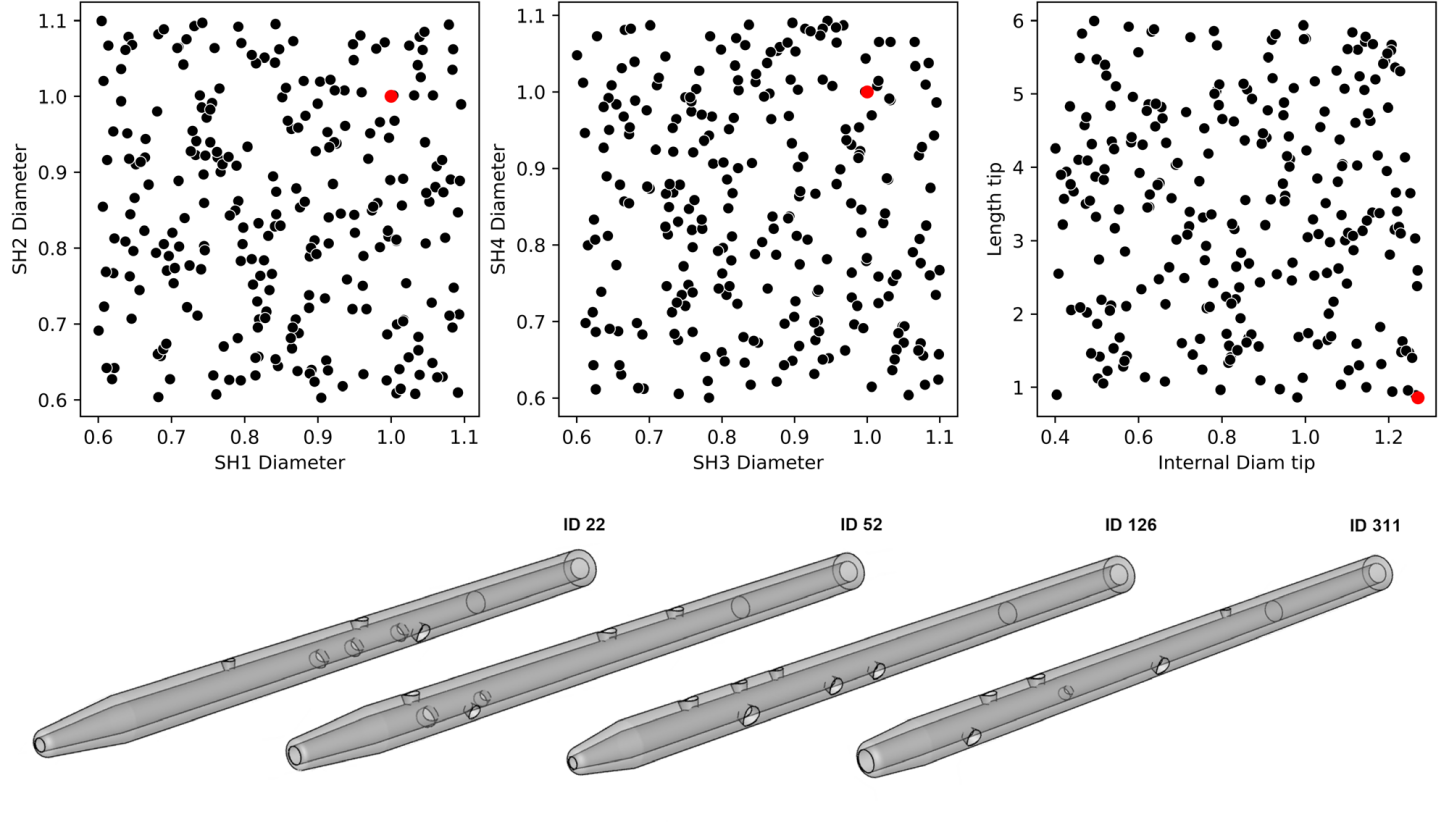
Sampling distribution and resulting models derived from the Tesio 6.5F catheter. Top: Overview of the sampling points distribution for the Tesio 6.5F-derived models plotted for selected input parameters: diameter of SH1, SH2, SH3, SH4, tip hole, and length of the distal tip hole. The red dot represents the original design. Bottom: example of Tesio 6.5F-derived models resulting from the sampling. SH, side hole.

The DOE was successfully run for the Tesio 6.5F (n = 699 simulations) and for the Hemo-Cath 8F (n = 389) models. Figure 2 shows the global samples distribution across the design space of the 6.5F for selected input parameters and four examples of designs.

### Computational Fluid Dynamics Analyses and Model Responses

**Figure 3.**
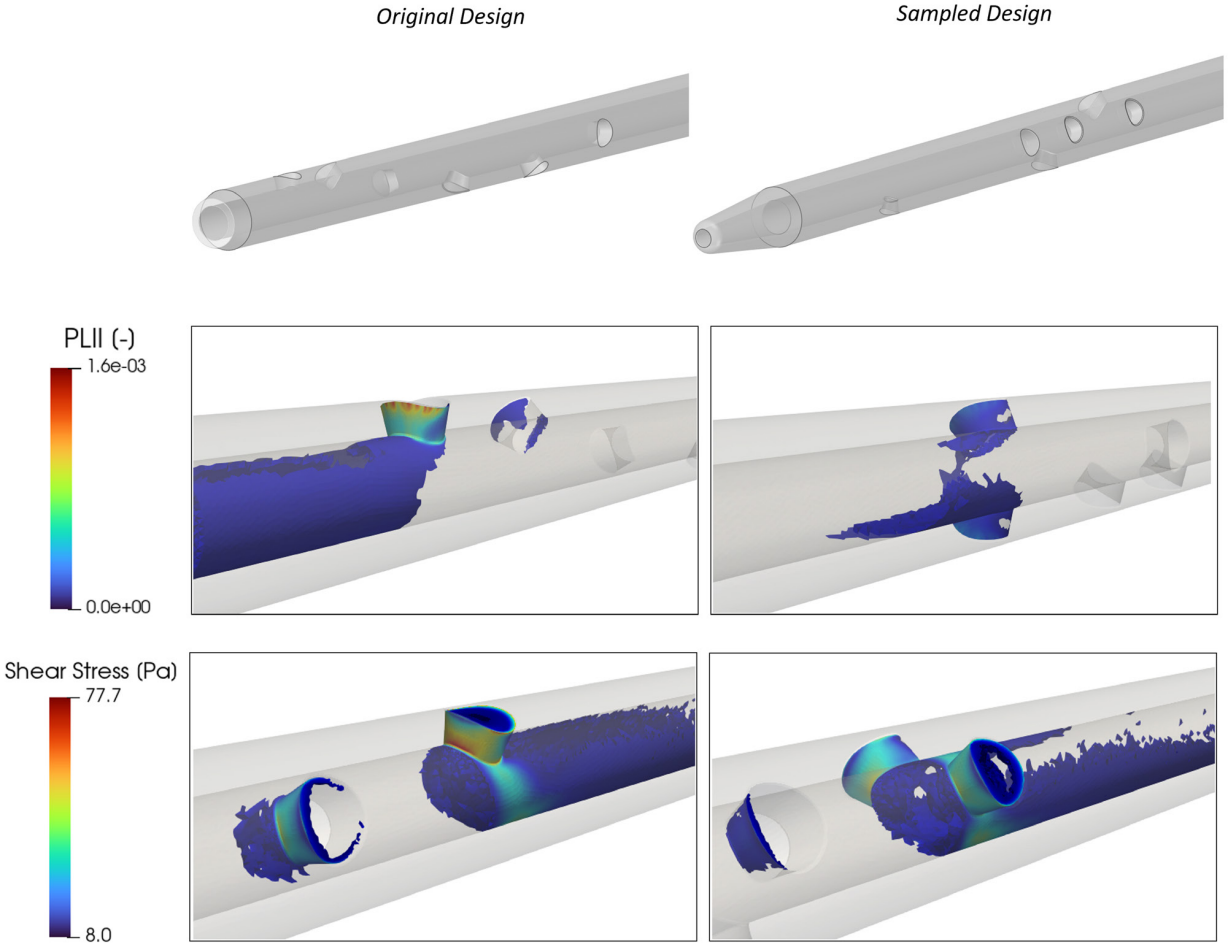
Overview of the CFD outputs for the Tesio 6.5F design and an example of a design from the sampling. The first row displays the geometries, while the second and third rows show the colormaps of PLI_l_ and shear stress, respectively, at the level of the most proximal side holes. CFD, computational fluid dynamics; PLI_l_, platelet lysis index.

Maps of PLI and SS are shown in Figure 3 for one of the designs derived from the Tesio 6.5F for a qualitative comparison which shows the impact of design choices on the parameters of interest.

Table 2 summarizes the CFD outputs for the design tested in this study. Increasing the flow rate imposed to the catheter increases the values of SS and PLI_l_ across all the samples (*p* ≪ 0.05). The RT does not change with the flow rate in the Tesio 6.5F-derived models (*p* = 0.29), most likely because of the poor flow going through the tip hole. The Hemo-Cath 8F derived models show reducing RT with increased flow rate (*p* ≪ 0.05) for both arterial and venous lumens, as expected.

**Table 2.** Average and SD of the CFD Outputs for the Population of Catheters (n) Derived From the Original Models at Three Different Working Flow Rates

CFD Outputs for All the Tested Designs									
	Tesio 6.5F			Hemo-Cath 8F (Arterial)			Hemo-Cath 8F (Venous)		
Blood Flow Rate (ml/min)	20	30	40	50	60	70	50	60	70
n	222	254	223	117	152	120	117	152	120
Max PLI_*l*_ (SD) (-)	0.7E-3 (0.0003)	1.9E-3 (0.001)	4.9E-3 (0.002)	1.2E-2 (0.005)	2.5E-2 (0.014)	3.7E-2 (0.018)	2.7E-2 (0.031)	5.2E-2 (0.049)	8.2E-2 (0.067)
Avg SS (SD) (Pa)	1.8 (0.3)	2.6 (0.5)	3.5 (0.7)	8.3 (1.2)	10.2 (1.5)	11.7 (1.7)	9.7 (0.9)	11.7 (1.0)	13.8 (1.1)
Avg RT (SD) (s)	0.28 (0.13)	0.29 (0.21)	0.28 (0.22)	0.11 (0.08)	0.09 (0.04)	0.09 (0.04)	0.05 (0.003)	0.04 (0.002)	0.03 (0.002)

CFD, computational fluid dynamics; PLI, platelet lysis index; RT, residence time; SD, standard deviation; SS, shear stress.

### Statistical Analysis on Tesio 6.5F Model

**Figure 4.**
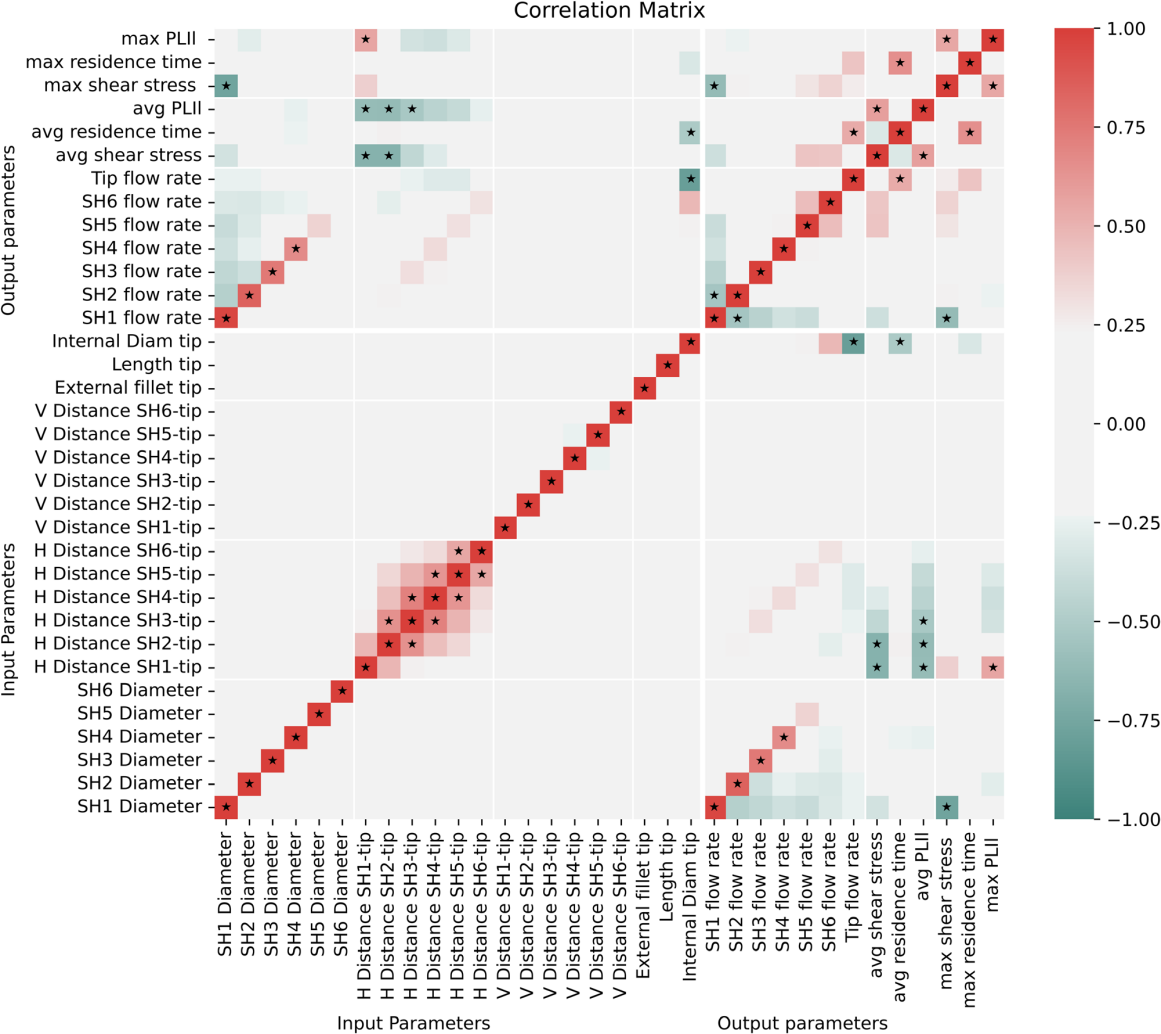
Correlation matrix for the arterial lumen of the Tesio 6.5 F model. A star is reported for the positive and negative correlations that are stronger than 0.50.

Figure 4 shows the correlation matrix for the arterial lumen of the Tesio 6.5F model.

#### Influence of side holes configuration

**Figure 5.**
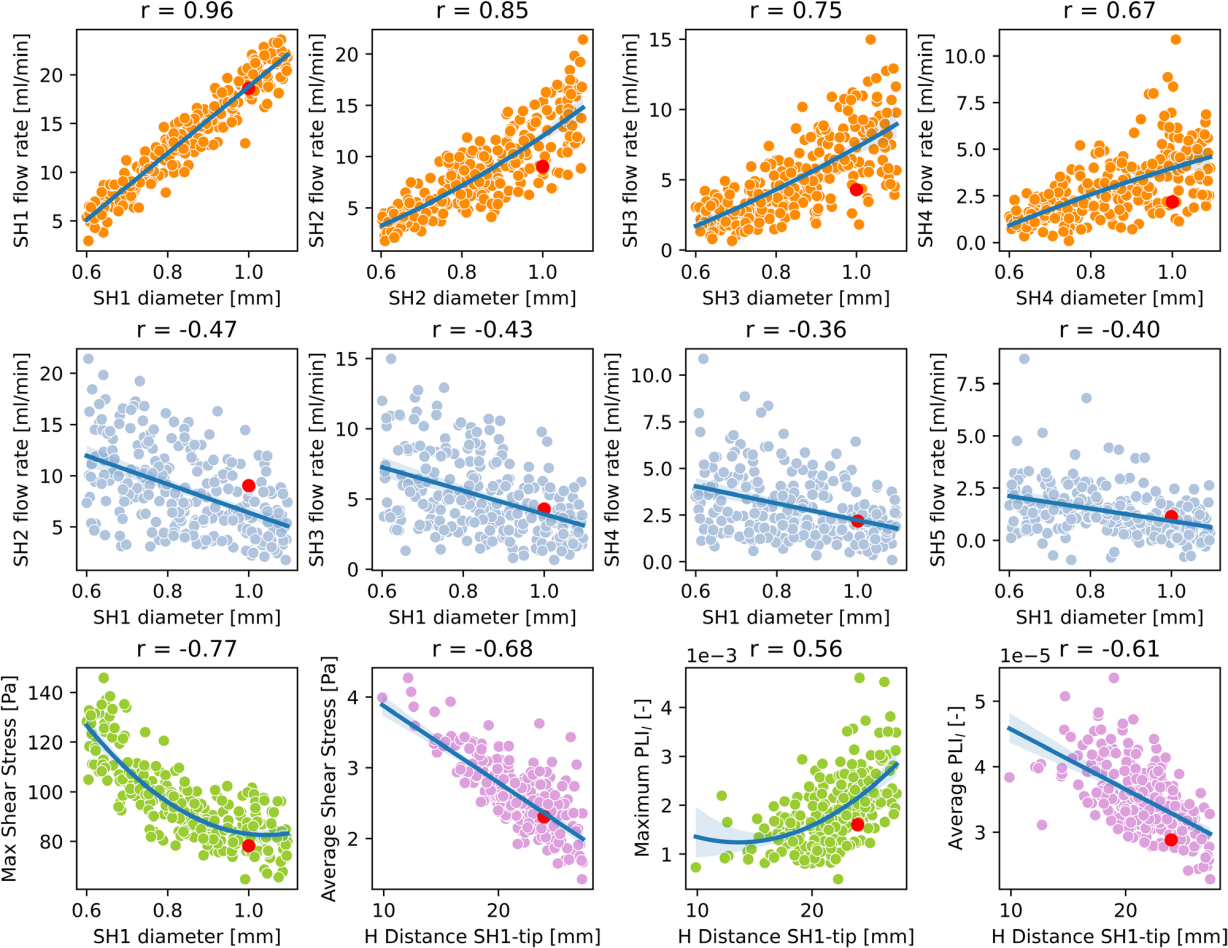
Scatter plots of selected correlations for the Tesio 6.5F model. Top row: flow rate of the first four SHs plotted against their diameters; central: flow rates of SHs 2, 3, 4, and 5 plotted against the diameter of SH 1; bottom row: SS and PLI_l_ (maximum and average) plotted against their most influential input parameters. The red dot represents the original design. PLI_l_, platelet lysis index; SH, side hole; SS, shear stress.

**Figure 6.**
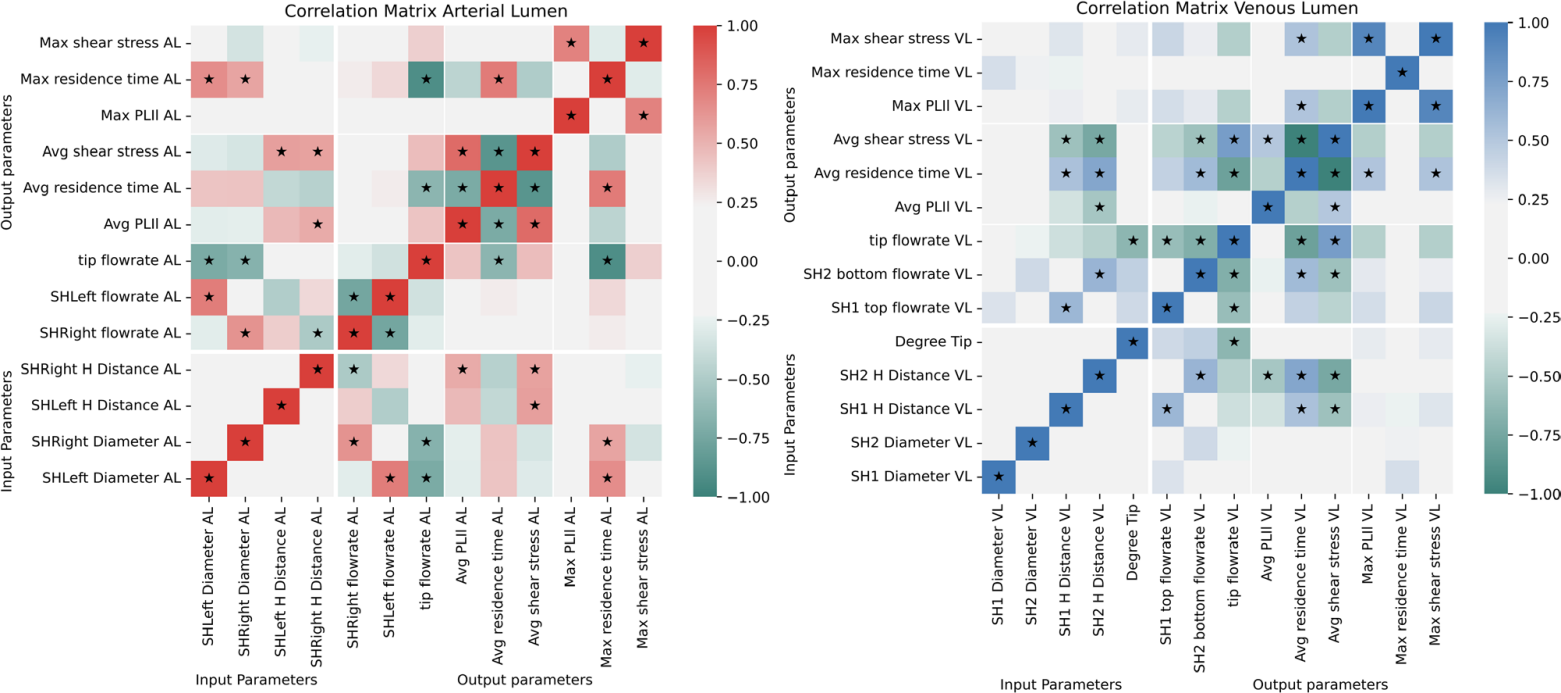
Correlation matrices for the arterial (right) and venous (left) lumens of the Hemo-Cath 8F. A star is reported for the positive and negative correlations that are stronger than 0.50. AL, arterial lumen; PLI_l_, platelet lysis index; SH, side hole; VL, venous lumen.

The correlation matrix for the Tesio 6.5F-derived models at 30 ml/min (Figure 5, top and central row) shows how the diameter of SH1, SH2, SH3, and SH4 correlated with the corresponding flow rate. An increase in the dimension of these holes resulted in higher blood flow entering the CVC (Figure 6, top row). This correlation was stronger in the first hole SH1 (*r* = 0.96) and weaker in the last hole SH6 (*r* = 0.14). The diameter of SH1 correlated negatively with the flow rates of the rest of the holes (Figure 6, central row). The diameters of SH2 and SH3 had a weak influence on the flow rate of the neighboring holes. Among the side holes, the diameter of SH1 also correlated negatively with the maximum SS (*r* = −0.77).

The axial distances between the side holes and the tip hole correlated negatively with the average values of SS and PLI_l_ at the tip of the catheter (Figure 5, bottom row). The influence was stronger for the most proximal holes (*i.e*., SH1, SH2, and SH3) with the correlation coefficients equal to −0.68, −0.68, and −0.43, respectively, when considering the SS, and −0.61, −0.62, and −0.53 for the PLI_l_.

The distance between SH1 and the tip hole showed moderate positive correlation with the maximum PLI_l_ (*r* = 0.56). The geometrical features of the side holes showed weak correlations with the residence.

#### Influence of the shape of the tip hole

The internal diameter of the tip hole showed strong and negative correlation (*r* = −0.81) with the flow rate of the tip hole and moderate and positive correlation (*r*=0.48) with the flow rate of SH6: increasing the diameter, the blood flow rate leaving the CVC increased. Accordingly, the average RT slightly reduced with the diameter of the tip (*r* = −0.51). Length of the distal tip and degree of the external fillet did not correlate with any outputs.

#### Influence of varying flow rates

As the flow rate of the CVC increased from 20 to 40 ml/min, the correlation between the distance of SH1 from the tip hole and the average PLI_*l*_ changed from weak at 20 ml/min (*r* = −0.13) to strong at 40 ml/min (*r* = −0.74). The influence of the diameter of SH1 on the maximum SS became stronger: *r* = −0.16 at 20 ml/min, *r* = −0.77 at 30 ml/min, and *r* = −0.95 at 40 ml/min. The correlation between the diameter of the tip hole and the flow rate of the tip decreased from *r* = −0.86 at 20 ml/min to *r* = −0.68 at 40 ml/min.

### Statistical Analysis on Hemo-Cath 8F Model

The correlation matrices at 60 ml/min for the arterial and venous lumens of the Hemo-Cath 8F model are shown in Figure 6.

#### Influence of side holes configuration

The correlation matrix for the arterial lumen (Figure 6, left) showed a symmetry between the right and left side holes of the catheter. Each side hole diameters correlated with its own flow rate (*r* = 0.73 for the left SH and *r* = 0.64 for the right SH), with the flow rate of the tip (*r* = −0.72 for the left side and *r* = −0.67 for the right side) and with the maximum RT (*r* = 0.66 for the left side and *r* = 0.56 for the right side). The distance between the tip entrance and the side holes correlated with flow rate of the side holes (*r* = −0.49 for the left side and *r* = −0.51 for the right side), average PLI_l_ (*r* = 0.47 and *r* = 0.54, for left and right side, respectively) and average SS (*r* = 0.58 for both sides). No correlation was found between any of the input parameters and average RT, maximum SS, and PLI_l_.

The correlation matrix of the venous lumen (Figure 6, right) showed how the diameter of the side holes did not correlate with flow rates nor affected the rest of the outputs. The distance between side holes and tip hole correlated to flow rate of the corresponding hole (*r* = 0.61 for the top hole and *r* = 0.62 for the bottom hole), average RT (*r* = 0.54 for the top hole and *r* = 0.72 for the bottom hole), and average SS (*r* = −0.57 for the top hole and *r* = −0.74 for the bottom hole).

The distance from the tip hole of the bottom hole showed a moderate correlation also with the average PLI_l_ (*r* = −0.54), this correlation was weaker on the left side (*r* = −0.35). The degree of opening of the tip hole correlated only with the tip flow rate (*r* = −0.65). The input parameters did not show significant correlation with the maximum values of SS, RT, and PLI_l_.

#### Influence of varying flow rates

As the flow rate of the CVC increased from 50 to 70 ml/min, the correlations increased mainly in the venous lumen in favor of the tip hole. In the arterial lumen, at flow rate increasing from 50 to 60 to 70 ml/min, the correlation between average RT and diameter of the two holes became stronger: *r* = 0.41, *r* = 0.50, and *r* = 0.58 for the right hole and *r* = 0.36, *r* = 0.47, and *r* = 0.51 for the left hole. In the venous lumen, the correlation between the flow rate through the tip hole and the distance between the top hole and the tip decreased (*r* = −0.42, *r* = −0.36, and *r* = −0.31) while increased the one with the degree of opening of the tip hole (*r* = −0.52, *r* = −0.64, and *r* = −0.72). The degree of opening of the tip hole showed higher correlation also with the flow rate of the top hole (*r* = 0.32, *r* = 0.36, and *r* = 0.48). At 70 ml/min, the maximum RT correlated moderately with the diameter of the top hole (*r* = 0.47 against *r* = 0.23 and *r* = 0.30 at 50 and 60 ml/min, respectively).

## Discussion

Central venous catheters are the most commonly used vascular access among pediatric HD patients. However, their failure rate in clinics remains high.^7,19–22^ Previous computational study showed flow criticalities potentially affecting their performance.^15^ Each CVC has multiple design features and operates at multiple flow conditions, depending on the size of the patient and the HD machines. Understanding the complex relationships between design and function is crucial to reduce the issues and improve the performance of such life-saving devices.

In our study, we used the DOE to evaluate the effects of specific design features on the performance of pediatric CVCs. Two models of CVCs, a 6.5F single-lumen model and an 8F double-lumen model, were selected for this study. To the best of our knowledge, this is the first time that a DOE was applied to pediatric CVCs.

For the designs derived from the Tesio 6.5F model, an imbalance among the role of the holes was found. Only the distance from the tip hole of the most proximal holes (SH1-3) correlated strongly with SS and the PLI_l_. The most proximal hole was also found to influence both the flow rates of the other openings and the maximum SS. Regarding the shape of the tip hole, only its internal diameter correlated with flow rate and RT potentially contributing to limit the areas of stagnation within the CVC. Interestingly, the analysis at three different flow rates enriched the landscape of the configurations tested amplifying the significance of the results.

In models derived from the Hemo-Cath 8F, the two arterial side holes showed similar influence on flow rates, maximum RT, average SS, and PLI_l_. On the venous lumen, the size of the holes did not impact the outputs. The distance of both holes from the tip correlated with average SS, RT, and PLI_l_, with the hole on the bottom being slightly more influential particularly on the PLI_l_.

The results of our study led us to the following practical design recommendations:

For the arterial lumen:

1. Four side holes placed at different positions along and around the catheter body should be sufficient to achieve the desired flow rate and provide alternative blood ports in case of obstruction due to vessel wall suction.
2. The most proximal side hole should be large enough to reduce maximum SS, average SS, and maximum RT, but without dominating the flow distribution.
3. The first two side holes should be positioned farther from the tip to reduce average SS and in parallel configuration to achieve equal contribution from the side holes.
4. The tip hole diameter should be large to guarantee quick washout and low RT.

For the venous lumen:

1. Side holes should be located farther from the tip to increase their flow rate and reduce SS, but this should be done carefully to avoid increasing RT.
2. The diameter of the tip opening should compromise between adequate flow rate and reduced RT.

Overall, the results of this study showed how the hemodynamics of pediatric catheters can be improved. A computational parametric approach able to test a wide number of catheters design can guide the creation of future optimized designs specifically for the pediatric population. In the field of vascular access for HD, there have been numerous innovations, but the solutions developed are largely piecemeal and far from perfect.^6^ The investment required for pediatric kidney disease is still poor: the number of pediatric-specific vascular access devices currently available is limited and data for access decisions are incomplete and frequently extrapolated from adult studies.^1,4^

## Limitations

This present study has some limitations: the large number of CFD simulations required for DOE were run in ideal geometries of SVC to reduce their computational cost. In addition, the CVLs were placed coaxially inside the vein and more realistic positions were not considered (*e.g*., CVL close to the wall of the vessel). The original design principles (*i.e*., lumen configuration, number, and shape of side holes) were not modified. This allowed to compare more directly the performance of the explored variations against the original, but it also limited the variety of designs explored. Finally, only the two smallest models of CVCs were considered. However, the methodology presented in this study can be applied to other CVC models in the future.

## Conclusions

In this study, the relationship between multiple design features and the performance of pediatric CVCs was analyzed by means of a DOE method. Results confirmed how side holes have a strong influence on the fluid dynamics, especially the most proximal ones when multiple holes are present. The role of the tip hole was found to be relevant and often conflicts with the one of the side holes. This analysis of the DOE represents the first step toward the optimization of CVCs. These findings will help to design new catheters for pediatric patients needing HD.
